# Supplementary figures and images for: Combining viral genomics and clinical data to assess risk factors for severe COVID-19 (mortality, ICU admission, or intubation) amongst hospital patients in a large acute UK NHS hospital Trust
Source: PLoS One. 2023 Mar 23;18(3):e0283447. doi: 10.1371/journal.pone.0283447 (PMC10035897; doi:10.1371/journal.pone.0283447)

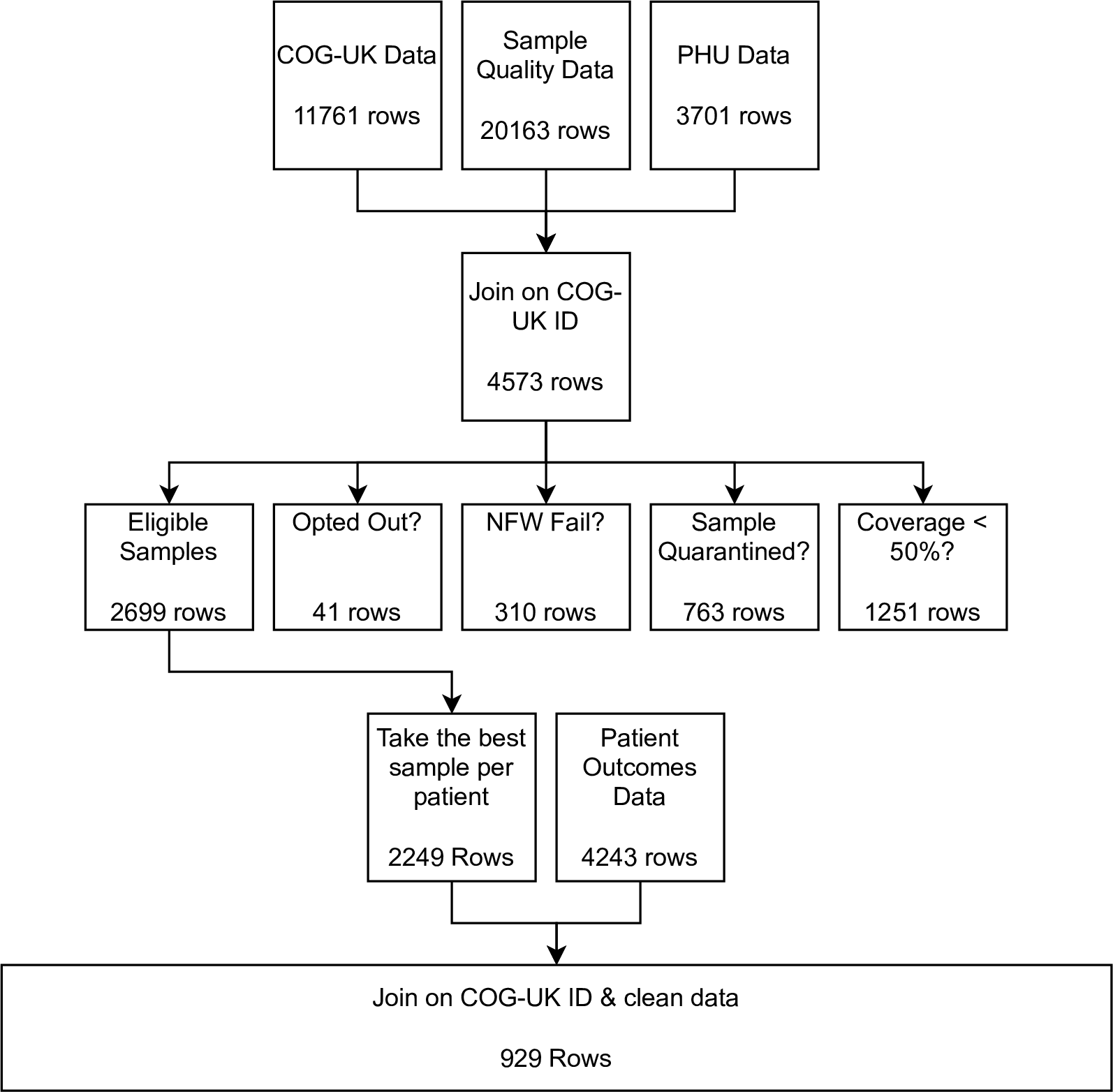

Supplement: S1 Fig — Flowchart of filtering steps for the final joint dataset. (TIF) [file pone.0283447.s001.tif]

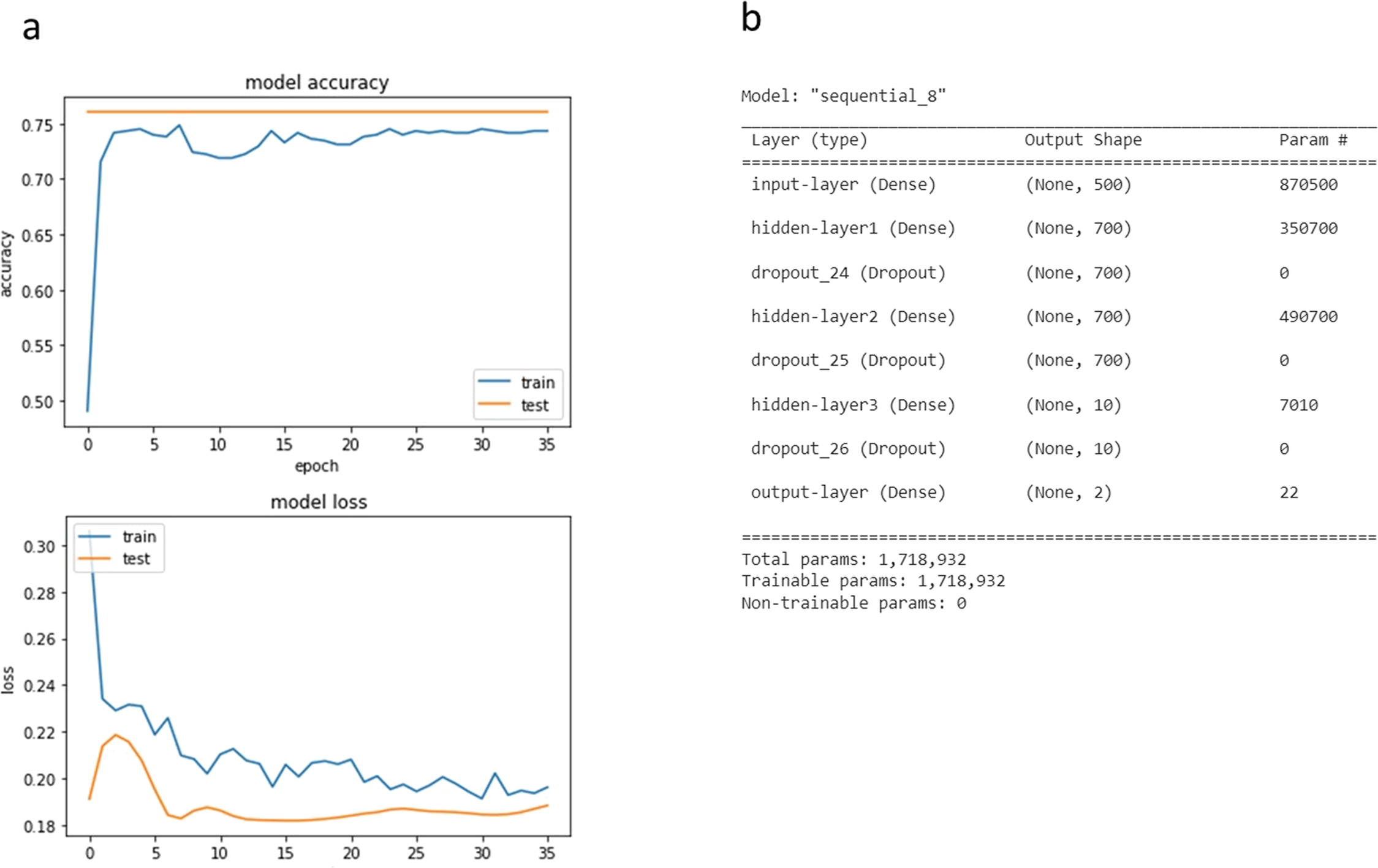

Supplement: S2 Fig — Performance metrics during the training and validation and the architecture of the MLP-ANN model. (a) The sequential method in Tensorflow v2.8 was used, incorporating the Adam optimization algorithm for stochastic gradient descent for training of deep learning models. Parameters used were a learning rate of 0.0001, with beta_1 = 0.9 and beta_2 = 0.799. Following initial stages of 10,000 epochs, the model was refined and optimised for the appropriate number of nodes and hidden layers, and an “early stopping” protocol was incorporated to stop training once the model performance stopped improving. This was determined using a concurrent evaluation of cross-validation loss remaining similar over 20 epochs, and ensured minimal over-fitting and improving computing time. The two graphs here show close convergence and agreement between the train and validation sets of the MLP-ANN model. (b) The final architecture of the MLP-ANN model. The model contained 3 hidden layers (with 700, 700 and 10 nodes each), and a final output layer containing two nodes to pipe the categorical outcomes of 0 (no-death) and 1 (death). The number of optimal nodes were optimised over several runs of model building and hyperparameter optimisation steps. The final layer used “softmax” as the activation step, which scales numbers/logits into probabilities. The activation steps for the hidden layers were ReLU, used specifically to address the problem of vanishing gradients in deep-learning models. Dropout regularization was employed to reduce overfitting of the model, where different sets of neurons are dropped from the architecture, giving an overall result akin to training and optimizing multiple neural networks simultaneously. (TIF) [file pone.0283447.s002.tif]

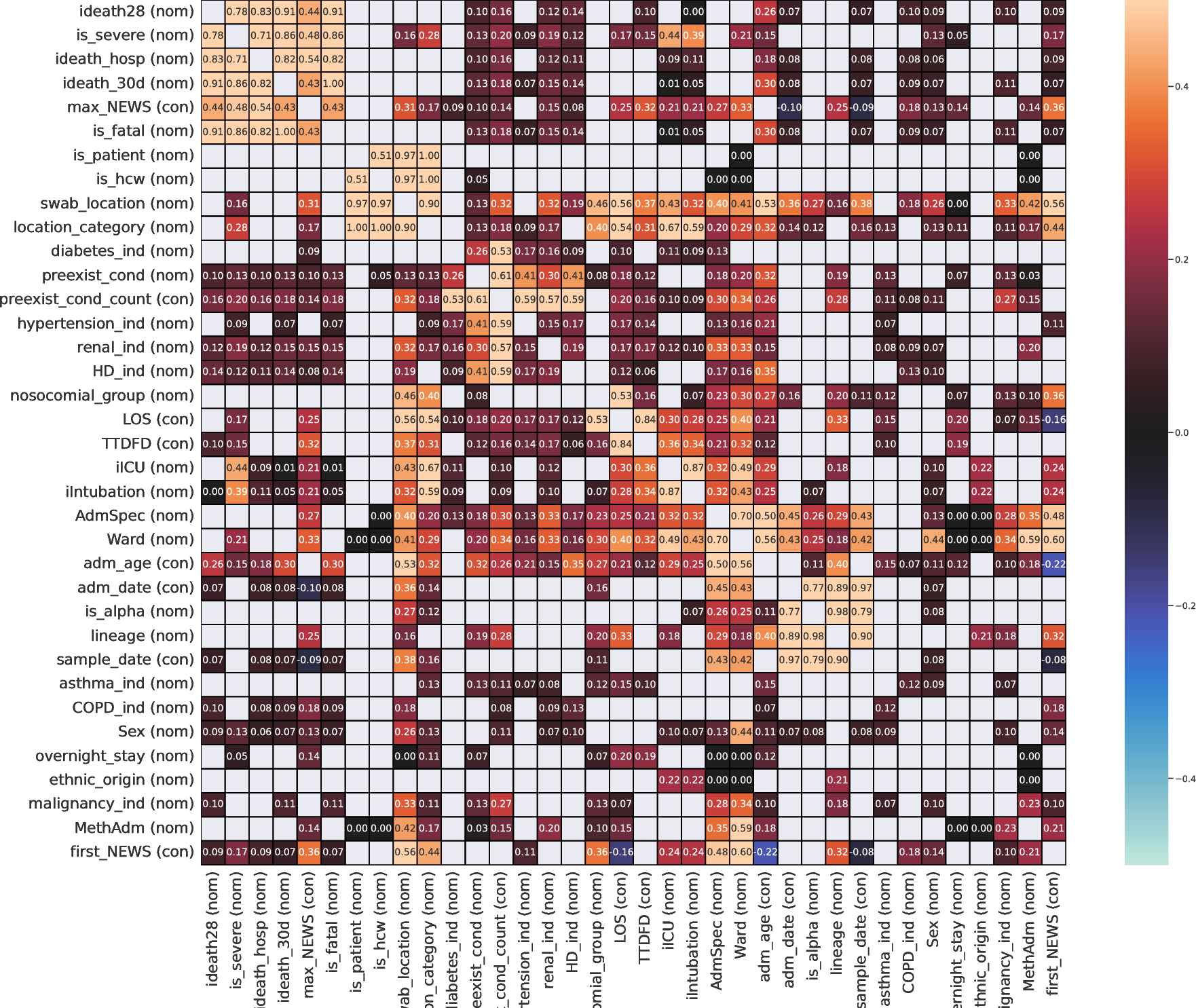

Supplement: S3 Fig — Heatmap showing pairwise association scores (based on Cramer’s V for categorical-categorical relationships, Spearman’s Rank for continuous-continuous relationships and Correlation Ratio for categorical-continuous relationships) between all variables in the patient outcomes dataset. Only statistically significant results are shown, with any association with p > 0.05 shown as grey. See S1 Table for description of data points. (TIF) [file pone.0283447.s003.tif]

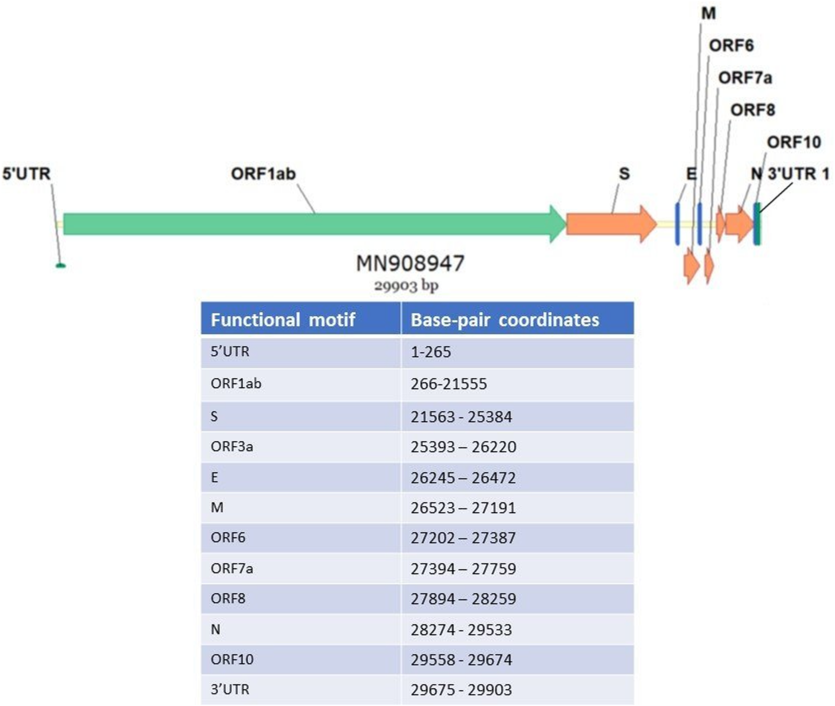

Supplement: S4 Fig — Schematic organisation of the SARS-CoV2 genome and the coordinates of nucleotide positions marking the boundaries of the various viral domains. The schematic diagram was generated using VectorNTI (V11). The wild-type SARS-CoV2 genome sequence was obtained from Genbank (Wuhan-Hu-1, GenBank, MN908947.3). The arrows indicate the direction of translation of the gene (5’ to 3’). (TIF) [file pone.0283447.s004.tif]
